# Acetylsalicylic acid in critically ill patients: a cross‐sectional and a randomized trial

**DOI:** 10.1111/eci.12771

**Published:** 2017-06-20

**Authors:** Christian Schoergenhofer, Eva‐Luise Hobl, Michael Schwameis, Georg Gelbenegger, Thomas Staudinger, Gottfried Heinz, Walter S. Speidl, Christian Zauner, Birgit Reiter, Irene Lang, Bernd Jilma

**Affiliations:** ^1^ Department of Clinical Pharmacology Medical University of Vienna Vienna Austria; ^2^ Department of Internal Medicine I Oncology & Hematology Medical University of Vienna Vienna Austria; ^3^ Department of Internal Medicine II Cardiology Medical University of Vienna Vienna Austria; ^4^ Department of Internal Medicine III Gastroenterology & Hepatology Medical University of Vienna Vienna Austria; ^5^ Clinical Institute of Laboratory Medicine Forensic Toxicology Unit Medical University of Vienna Vienna Austria

**Keywords:** Acetylsalicylic acid, critically ill, pharmacokinetics, platelet aggregation, thromboxane

## Abstract

**Background:**

Despite decades of clinical use, the pharmacokinetics and the effects of acetylsalicylic acid (ASA) in critically ill patients remain ill‐defined. We aimed to investigate the pharmacokinetics and the effects of different ASA formulations during critical illness.

**Design:**

A cross‐sectional study and a randomized, parallel‐group trial were performed. Critically ill patients under chronic oral ASA treatment (100 mg enteric‐coated) were screened for high ‘on‐treatment’ platelet reactivity (HTPR) according to arachidonic acid‐induced whole‐blood aggregometry. Thirty patients with HTPR were randomized to receive 100 mg ASA intravenously, 100 mg enteric‐coated ASA bid (bis in die) or 81 mg chewable ASA (*n* = 10 per group). Serum thromboxane B2 (TXB2) levels, ASA and salicylic acid levels were quantified.

**Results:**

Of 66 patients, 85% (95% confidence intervals 74–93%) had HTPR. Compared to baseline infusion of 100 mg, ASA significantly reduced platelet aggregation after 24 h to median 80% (Quartiles: 66–84%). Intake of 81 mg chewable ASA significantly reduced platelet aggregation to 75% (54–86%) after four hours, but increased it to 117% after 24 h (81–163%). Treatment with 100 mg enteric‐coated ASA bid decreased platelet aggregation after 24 h to median 56% (52–113%). Baseline TXB2 levels were median 0·35 ng/mL (0·07–0·94). Infusion of ASA or intake of 100 mg ASA bid reduced TXB2 levels to 0·07–0·18 ng/mL after 24 h, respectively. Chewable ASA reduced TXB2 levels only transiently. Pharmacokinetic analysis revealed highly variable absorption patterns of oral ASA formulations.

**Conclusion:**

There is a very high prevalence of HTPR in critically ill patients on peroral ASA therapy, caused by an incomplete suppression of TXB2 and/or by impaired absorption of ASA.

## Introduction

For decades, acetylsalicylic acid (ASA) has been a mainstay in the secondary prophylaxis of cardiovascular diseases 1, whereas its role in primary prevention remains debatable 2. Intake of low‐dose ASA was associated with improved outcome in critically ill patients 3, in patients with *Staphylococcus aureus* bloodstream infections 4 or in patients with septic shock from community‐acquired pneumonia 5.

High on‐treatment platelet reactivity (HTPR) is defined as an insufficient inhibition of platelets despite antiplatelet therapy that may be associated with an increased risk of experiencing cardiovascular events 6. In contrast to P2Y12 inhibitors, no well‐defined thresholds exist for treatment with ASA 6. Depending on cut‐offs and platelet function tests, the prevalence of HTPR ranges between ~20% and 30% 7, 8.

Surprisingly, no data are available on the prevalence of HTPR or on the pharmacokinetics of ASA in critically ill patients. However, the pharmacological properties of drugs during critical illness may be significantly altered, that is due to impaired gastric motility, altered bioavailability after enteral administration, altered drug distribution, organ dysfunction or altered enzyme activity 9, 10, 11. Furthermore, critically ill patients may have an increased platelet turnover with increased risk of HTPR 12.

We hypothesized that HTPR is frequent in critically ill patients because of altered pharmacokinetics and systemic inflammation. The aim of this trial was to investigate the drug concentrations and the antiplatelet effects of low‐dose ASA in critically ill patients. Furthermore, we investigated whether a second dose, an intravenous infusion or an alternative formulation of ASA, improves the response to ASA in poor responders to standard ASA treatment.

## Materials and methods

The Department of Clinical Pharmacology of the Medical University of Vienna coordinated the trial and included patients admitted to medical ICUs of the General Hospital of Vienna. The independent Ethics Committee of the Medical University of Vienna and the competent authorities approved the trial, which was conducted in full commitment with the Declaration of Helsinki and the Good Clinical Practice guideline. The project was registered at clinicaltrials.gov and at EudraCT with the identifiers NCT02285751 and 2012‐002226‐76, respectively. Informed consent was sought from all patients before inclusion. However, in patients unable to give informed consent at the time of inclusion, the ethics committee waived consent. The protocol was uploaded as a supplement. Reporting of the study conforms to CONSORT‐revised along with references to CONSORT‐revised and the broader EQUATOR guidelines 13.

### Patients

Inclusion criteria requested patients > 18 years of age, admitted to a medical ICU with pre‐existent low‐dose ASA treatment [oral 100 mg/day enteric‐coated ASA (Thrombo‐ASS, Gerot Lannach Pharma, Lannach, Austria)]. Exclusion criteria included allergies or hypersensitivities to the trial drugs, active bleeding, known coagulation disorders or intake of other antiplatelet drugs.

### Noninterventional trial

Blood samples were drawn before the daily dose of ASA was administered, orally or via a nasogastric tube, 2 and 24 h thereafter. ASA tablets had to be crushed and/or dissolved in 0·9% sodium chloride solution, if they were administered via a nasogastric tube.

### Interventional trial

In this open‐label trial, thirty patients diagnosed with HTPR were randomized to receive 81 mg chewable ASA (Bayer chewable aspirin, Morristown, NJ, USA, *n* = 10), 100 mg intravenous ASA (Aspisol, Bayer Schering Pharma, Berlin, Germany, *n* = 10) or 100 mg enteric‐coated ASA bis in die (bid, twice daily) at 8:00 a.m. and 8:00 p.m. (*n* = 10) for 1 day. Blood sampling was performed 1, 2, 4 and 24 h after the respective treatment was administered.

### Platelet function assays

To assess platelet function, we performed arachidonic acid (AA)‐induced whole‐blood aggregometry and measured platelet function under high shear rates, both standard methods to assess responsiveness to ASA treatment 14.

Whole‐blood aggregation was determined using the multiple electrode aggregometry (MEA) on the Multiplate Analyzer (Dynabyte Medical, Munich, Germany). AA‐ and ADP‐induced platelet aggregation were performed as explained previously 15 (Appendix S1). Based on other trials, we chose a cut‐off of > 30 U (arbitrary units) 16, 17, 18.

The platelet function analyzer‐100 (PFA‐100; Dade Behring, Marburg, Germany) was used for measuring platelet function under high shear rates (5000–6000 s^−1^) as described previously 19 (Appendix S1). Collagen/epinephrine (PFA‐EPI) and collagen/ADP (PFA‐ADP) coated cartridges were used. As a cut‐off value for HTPR, a closure time < 193 s for PFA‐EPI was recommended 20.

Thromboxane B2 (TXB2) levels were measured by enzyme‐linked immunoassay (ELISA; Cayman Chemical, Ann Arbor, MI, USA) as previously reported 20.

### Pharmacokinetics

Plasma concentrations of ASA and salicylic acid were determined by liquid chromatography tandem mass spectrometry (LC‐MS/MS) based on a published procedure (Appendix S1) 21.

### Disease scores

The Sequential Organ Failure Assessment (SOFA) score and the simplified acute physiology score (SAPS) III were calculated on trial day 1 22, 23.

### Randomization

Physicians enrolled patients, who were assigned continuous identification numbers. Patients with HTPR on trial day 1 were available for randomization. Physicians or a trained study nurse randomized patients through an online randomization programme (https://www.meduniwien.ac.at/randomizer/web/login.php) using permuted block randomization.

### Statistics

No data were available on the prevalence of HTPR in ICU patients. In stable patients, a HTPR rate of 20–30% was reported 7, 8. To randomize 30 patients, we assumed that we need to screen a multiple of that and initially planned to include at least 100 patients. However, due to the high rate of HTPR in critically ill patients we finished the trial after inclusion of 66 patients.

We estimated a mean area under the curve (AUC) of 30 U in AA‐induced platelet aggregation in patients with HTPR 18. Thus, a sample of *n* = 10 per group allowed us to detect a significant reduction in platelet function from an AUC of 30 to 10 with a power of > 90% and a corrected alpha error of 1·6%. HTPR rates are presented as percentage ± 95% confidence intervals (CI).

The primary endpoint was AA‐induced platelet aggregation. Secondary endpoints included drug concentrations, platelet function under high shear rates, TXB2 concentrations and correlations.

A repeated‐measures anova was performed to compare repeatedly measured parameters (in detail: Appendix S1). To adjust for multiple comparisons, we performed the Bonferroni procedure.

For exploratory analysis, pairwise comparisons were performed by the Wilcoxon test, and unpaired comparisons with the Kruskal–Wallis anova followed by a Mann–Whitney *U*‐test. Due to the exploratory character of these analyses, no corrections for multiple testing were applied. Correlations were calculated using the nonparametric Spearman test.

## Results

**Figure 1:**
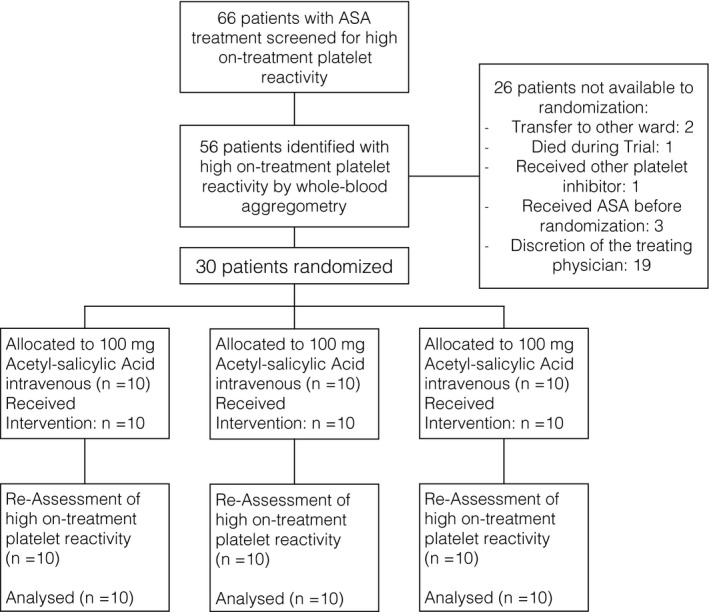
Flow chart of the trial.

Sixty‐six critically ill patients were recruited and finished the trial between 13 November 2012 and 25 March 2016 (Fig. 1). The trial ended after the last visit of the last patient. Patient demographics and baseline data are presented in Table 1. The 28‐day mortality was 38%, which corresponded well with the predicted mortality from the simplified acute physiology score 3 22. All randomized patients completed the trial per protocol, and data were available for all analyses, while in the observational part, 24‐h values of nine subjects were missing (Fig. 1).

**Table 1:** Demographics and baseline data

Parameter	Mean ± Standard deviation^a^
Gender m (f)	51 (15)
Age (years)	67 ± 13
Height (cm)	174 ± 8
Weight (kg)	85 ± 20
Haemoglobin (g/dL)	9·8 ± 1·4
Platelets (10^9^)	200 ± 100
Leucocytes (10^9^)	11·1 ± 4·3
C‐reactive protein (mg/dL)	12·0 ± 9·1
SAPS 3 score trial day (points)	61 ± 14
SOFA score (points)	7 ± 4
Time to inclusion (days)	9·8 ± 10·7
Admission diagnosis	
Cardiac	18 (27%)
Cardiopulmonary resuscitation	21 (32%)
Infection	10 (15%)
Respiratory disease	10 (15%)
gastrointestinal disease	1 (2%)
thromboembolism (i.e. Stroke, Pulmonary embolism)	3 (5%)
Others	3 (5%)
Catecholamine treatment *n* (%)	20 (30%)
Continuous veno‐venous hemodiafiltration *n* (%)	12 (18%)
Nonsteroidal anti‐inflammatory drugs	21 (32%)
Lactate (mM)	1·0 ± 0·4

a Presented are means ± standard deviations and numbers (%) of all 66 patients.

### Platelet aggregation

**Figure 2:**
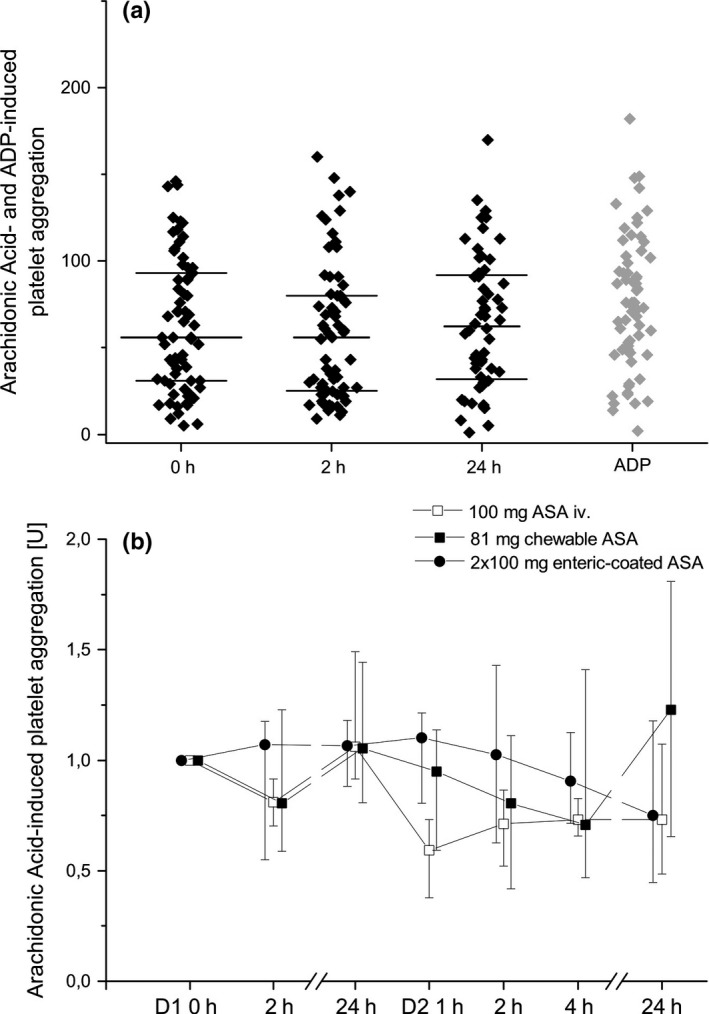
Arachidonic acid‐ and ADP‐induced platelet aggregation using whole‐blood aggregometry. Panel (a) Arachidonic acid‐induced platelet aggregation during screening, individual values (dots), median and quartiles (horizontal lines) Panel (b) Relative changes in arachidonic acid‐induced platelet aggregation at baseline and after 100 mg intravenous, 100 mg enteric‐coated acetylsalicylic acid (ASA) bid or 81 mg chewable ASA, presented are medians ± interquartile ranges; 0 h or D1 0 h = before intake or infusion of Aspirin, 1 h = 1 h after intake or infusion, 2 h = 2 h after intake or infusion, 4 h = 4 h after intake or infusion, 24 h = 24 h after intake or infusion of ASA.

Fifty‐six of 66 patients (85%, 95% CI: 74–93%) had HTPR according to the defined criteria: > 30 U in the AA‐induced platelet aggregation (Fig. 2). Continued intake of 100 mg enteric‐coated ASA transiently decreased platelet aggregation after 2 h (*P* = 0·038 vs. 0 h). Aggregation was similar between 0 and 24 h.

Platelet aggregation between the three groups did not differ significantly. However, platelet aggregation changed significantly over time (*P* < 0·001). This indicates the impact of each intervention on platelet aggregation during 24 h.

Infusion of 100 mg ASA i.v. and intake of 81 mg chewable ASA significantly reduced AA‐induced platelet aggregation (*P* = 0·007 and *P* = 0·001, univariate analysis, Table S1). However, intake of 100 mg enteric‐coated ASA bid did not significantly reduce platelet aggregation in univariate analysis; numerically MEA values after 24 h were the lowest of all groups (Table S1). The impact of the alternative treatments on HTPR is presented in Table S3.

Results of AA‐ and ADP‐induced platelet aggregation correlated well at baseline (*r* = 0·64 *P* < 0·001). Both tests also correlated reasonably well with the platelet count (AA: *r* = 0·49, *P* < 0·001; ADP: *r* = 0·7, *P* < 0·001). However, platelet aggregation did not correlate (not significant or *r* < 0·3) with haemoglobin level, fibrinogen, serum C‐reactive protein level or disease scores (SOFA, SAPSIII).

### Platelet function under high shear rates

Twenty‐nine of 66 patients (44%, 95% CI: 32–57%) were diagnosed with HTPR according to the defined criteria: closure time < 193 s for epinephrine‐coated cartridges.

PFA‐EPI and PFA‐ADP showed only a weak correlation at baseline (*r* = 0·34, *P* = 0·005). PFA‐ADP correlated weakly with platelet count (*r* = 0·37, *P* = 0·003). PFA‐EPI or PFA‐ADP did not correlate (not significant or *r* < 0·3) with haemoglobin level, leucocyte count, fibrinogen, C‐reactive protein level or disease scores (SOFA, SAPSIII).

### TXB2 results

**Figure 3:**
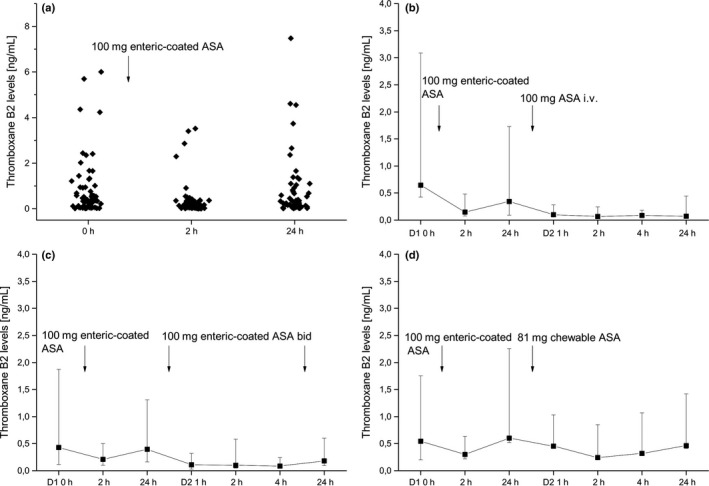
Plasma thromboxane B2 (TXB2) concentrations in critically ill patients treated with 100 mg enteric‐coated acetylsalicylic acid (ASA) (panel a), 100 mg intravenous ASA (panel b, *n* = 10), 100 mg enteric‐coated ASA bid (panel c, *n* = 10) and 81 mg enteric‐coated ASA (panel d, *n* = 10). Presented are medians ± interquartile ranges.

At baseline, before the next daily dose of 100 mg enteric‐coated ASA, median TXB2 levels were 0·35 (quartiles: 0·07–0·94) ng/mL. Intake of 100 mg enteric‐coated ASA reduced TXB2 levels to 0·12 (0·05–0·33) ng/mL after 2 h *P* < 0·001 vs. 0 h), which almost returned to baseline 0·25 (0·08–0·82) ng/mL 24 h thereafter (Fig. 3).

In multivariate testing, there was no significant difference between the three treatment groups. Only intake of 100 mg enteric‐coated ASA bid reduced TXB2 levels significantly over time (univariate analysis, *P* = 0·048).

Infusion of ASA reduced TXB2 levels from a baseline 0·35 (0·25–1·38) ng/mL to median 0·07–0·1 ng/mL for the next 24 h (pairwise comparisons, *P* = 0·007, *P* = 0·005, *P* = 0·005, *P* = 0·017, for baseline vs. 1 h, 2 h, 4 h, 24 h, Fig. 3). Intake of 100 mg enteric‐coated ASA bid decreased TXB2 levels from median 0·4 (0·24–0·92) ng/mL at baseline to median 0·09–0·18 ng/mL (*P* = 0·021, *P* = 0·13, *P* = 0·026, *P* = 0·05, for baseline vs. 1 h, 2 h, 4 h, 24 h) for the next 24 h. Treatment with 81 mg chewable ASA tablets reduced TXB2 levels from median 0·6 ng/mL (0·08–1·65 ng/mL) at baseline to median 0·25–0·46 ng/mL (*P* = 0·005, *P* = 0·005, *P* = 0·09, *P* = 0·88, for baseline vs. 1 h, 2 h, 4 h, 24 h) in the next 24 h.

We recorded the intake of nonsteroidal anti‐inflammatory drugs, which was equally distributed between the randomized groups (2 or 3 per group) and had no apparent effect on TXB2 measurements.

### Pharmacokinetics

There were no significant differences between the three treatment groups in multivariate analysis, although drug concentrations changed significantly over time (*P* < 0·001).

**Figure 4:**
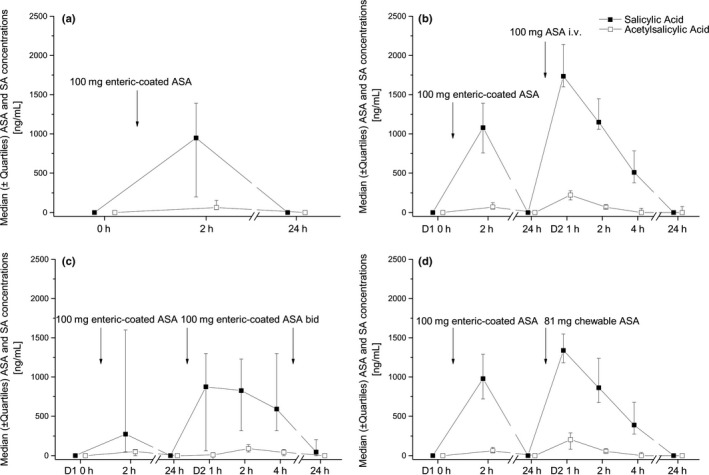
Plasma concentrations of acetylsalicylic acid (ASA) (□) and salicylic acid (■) of 100 mg enteric‐coated ASA (left upper quadrant, *n* = 66), 100 mg enteric‐coated ASA bid (right upper quadrant, *n* = 10), 100 mg ASA i.v. (left lower quadrant, *n* = 10) and 81 mg chewable ASA (right lower quadrant, *n* = 10). Medians ± interquartile ranges are presented.

Peak concentrations of ASA (*P* = 0·013) and salicylic acid (*P* = 0·005) were higher after infusion of 100 mg ASA compared to 100 mg enteric‐coated ASA and maximum salicylic acid levels compared to 81 mg chewable ASA (*P* = 0·023). Plasma concentrations of ASA and salicylic acid were similar between 81 mg chewable ASA and 100 mg enteric‐coated ASA. Plasma concentrations of ASA and salicylic acid levels are presented in Table S2 and Fig. 4.

The coefficient of variation of salicylic acid concentrations was 80% 2 h after administration of 100 mg enteric‐coated ASA, 70% after intake of 81 mg chewable ASA and 45% after infusion of 100 mg ASA.

The *T*_1/2_ of salicylic after infusion of ASA was approximately 102 and 96 min after intake of chewable ASA. The *T*_1/2_ of enteric‐coated ASA could not be determined because of slow absorption.

### Adverse events

The trial lasted 24 h in 36 patients and 48 h in 30 patients. No drug‐related adverse events occurred in the interventional trial. Two patients experienced major bleeding and one patient acute cerebral infarction in the noninterventional study. One patient died during the noninterventional trial.

## Discussion

Our main findings are that (i) the prevalence of HTPR is much higher in critically ill patients than reported previously 7, 8, (ii) the plasma concentrations of ASA and salicylic acid are highly variable, and (iii) TXB2 levels, although suppressed to a large extent, show a remaining potentially pathophysiologically relevant background activity in many patients.

In patients with acute myocardial infarction with chronic ASA therapy, TXB2 levels were 0·09 ng/mL (quartiles: 0·03–0·43) 24. Additional infusion of 250 mg ASA decreased TXB2 levels to 0·04 ng/mL (0·01–0·04 ng/mL) and significantly lowered HTPR 24. This indicates that thromboxane levels < 0·43 ng/mL are still relevant. Median TXB2 concentrations in our population were 0·35 ng/mL and thus ~fourfold higher 24. Furthermore, a discrepancy between the measurement of serum TXB2 *ex vivo* and thromboxane generation *in vivo*, assessed by excretion of 2,3‐dinor‐thromboxane, was reported 25. In healthy volunteers, a 94 ± 1% inhibition of serum TXB2 *ex vivo* by intake of aspirin only translated to a 28 ± 8% inhibition of urinary 2,3‐dinor‐thromboxane *in vivo* 25. However, the *in vivo* generation of thromboxane was reduced by increasing the degree of serum TXB2 inhibition to ≥ 95% 25. Thus, ≥ 95% inhibition of TXB2 could be necessary to achieve sufficient platelet inhibition.

Surprisingly, even after the infusion of ASA, TXB2 was not fully suppressed. Although thromboxane is generally regarded as COX‐1 dependent 26, other sources of thromboxane generation have been reported 27, 28 including COX‐2‐dependent thromboxane generation during inflammatory states 29 or in reticulated platelets 30. Moreover, nucleated cells may regenerate COX‐1 31. Treatment of septic patients with ibuprofen had beneficial effects on metabolic biomarkers, such as oxygen consumption or blood lactate levels, and it reduced the excretion of thromboxane metabolites, but the mortality benefit did not reach significance (37% vs. 40%) 32. However, this trial included 455 patients and was only powered to detect a 35% difference in mortality between groups. The observed difference would have required a sample size of approximately > 8·000 patients.

We detected a surprisingly high HTPR rate. Most studies report a HTPR rate of approximately 20% using MEA 33. In patients undergoing haemodialysis, 50% of patients had HTPR 34. Interestingly, in patients undergoing coronary artery bypass‐graft surgery, the HTPR rate increased postoperatively 17, 35. Various factors, such as platelet count, haematocrit, leucocyte count, red blood cell count, C‐reactive protein levels or fibrinogen, correlated with the results of MEA 16, 36. In our trial, only platelet count correlated well with MEA results. However, our population was not homogenous and probably too small to find such associations. The extraordinary high rate of HTPR in our trial may be explained by the residual thromboxane activity, a higher platelet turnover and possibly an impaired absorption after oral ASA treatment.

The PFA‐100 classified 44% of patients with HTPR, which is in line with other reports 37. The closure time detected by this system is affected by haematocrit, platelet count and the von Willebrand factor activity 38. There is limited knowledge on its applicability in critically ill patients. Platelet function measurements with CADP cartridges showed a generalized platelet defect in 24 of 66 patients. In these patients, detection of HTPR may not have been possible, and the prevalence of HTPR may therefore be underestimated.

Crushing and/or dissolving tablets affects the pharmacokinetics with a shorter *T*_max_ compared to intake of whole tablets 39. Individual plasma concentration–time curves after intake of oral ASA formulations suggest highly variable absorption patterns in critically ill patients (Figure S1), which is also reflected by high coefficients of variation for salicylic acid levels ranging between 45% and 80% 2 h after intake. In some patients, drug absorption seems to be delayed possibly due to reduced gastrointestinal motility. Compared to healthy volunteers, the salicylic acid concentrations after intake of ASA were markedly lower in our patients, regardless of the formulation 18, 39. Thus, absorption of orally administered drugs is severely impaired in some critically ill patients. Therefore, especially in high‐risk patients, intravenous formulations should be considered to optimize bioavailability.

The half‐life of salicylic acid on day 2 for i.v. and chewable ASA was approximately 100 min. This is in line with other studies 18 and demonstrates that metabolism and excretion of ASA is not altered in ICU patients.

Taken together, these data indicate that in order to ensure adequate platelet inhibition in critically ill patients, clinicians should consider using intravenous formulations, and/or possibly two daily doses. Treatment with oral formulations bears the risk of insufficient absorption of ASA and consequently reduced antiplatelet effects.

### Limitations

The included population was quite inhomogeneous. However, all patients were treated with ASA and we aimed to define the prevalence of HTPR in ICU patients regardless of the underlying disease. Secondly, this study was powered to show improvements in platelet function assays by alternative treatments and to show the significantly increased rate of HTPR in critically ill patients, but not to assess the clinical impact of HTPR in these patients. Thirdly, we were only able to conduct sparse pharmacokinetic analyses due to the severe condition of patients. Finally, this trial did not interfere with regular treatment of patients and the intake of nonsteroidal antiphlogistic drugs was therefore not forbidden.

## Conclusion

The measured TXB2 levels and the high HTPR rate suggest insufficient platelet inhibition by ASA in critically ill patients. This may in part be caused by the impaired absorption of orally administered drugs or by COX‐2‐dependent thromboxane generation during systemic inflammation. Intravenous formulations reduce the substantial variability of ASA/salicylic acid concentrations and could be a better choice for high‐risk patients in the ICU.

## Disclosures

The authors report no conflict of interests.

## Sources of funding

The Austrian Science Funds (FWF, grant number SFB54P04) funded this work. The funding source played no role in study design, in the collection, analysis or interpretation of data, in the writing of the report, and in the decision to submit the article for publication.

## Authors’ contribution

CS and BJ designed the trial. All authors were involved in the conduct of the trial and in the acquisition of data. EH and BR measured drug levels. All authors were involved in the analysis and interpretation of the trial. CS and BJ drafted the manuscript. All authors critically revised the manuscript and approved the final version for publication.

## Address

Department of Clinical Pharmacology, Medical University of Vienna, Währinger Gürtel 18‐20, 1090 Vienna, Austria (C. Schoergenhofer, E.‐L. Hobl, M. Schwameis, G. Gelbenegger, B. Jilma); Department of Internal Medicine I, Oncology & Hematology Medical University of Vienna, Währinger Gürtel 18‐20, 1090 Vienna, Austria (T. Staudinger); Department of Internal Medicine II, Cardiology Medical University of Vienna, Währinger Gürtel 18‐20, 1090 Vienna, Austria (G. Heinz, W. S. Speidl, I. Lang); Department of Internal Medicine III, Gastroenterology & Hepatology, Medical University of Vienna, Währinger Gürtel 18‐20, 1090 Vienna, Austria (C. Zauner); Clinical Institute of Laboratory Medicine, Forensic Toxicology Unit, Medical University of Vienna, Währinger Gürtel, 18‐20, 1090 Vienna, Austria (B. Reiter).

## Supporting information


**Appendix S1.** Methods

**Figure S1.** Individual plasma concentrations of acetylsalicylic acid and salicylic acid in ng/mL after intake of 100 mg enteric‐coated acetylsalicylic acid (left upper panel), 81 mg chewable acetylsalicylic acid (right upper panel), 100 mg enteric‐coated acetylsalicylic acid (left lower panel) and infusion of 100 mg acetylsalicylic acid (right lower panel).

**Table S1.** Arachidonic Acid‐induced platelet aggregation using whole blood aggregometry after alternative ASA treatments.

**Table S2.** Acetylsalicylic acid (ASA) and Salicylic acid (SA) plasma concentrations after different ASA treatments.

**Table S3.** HTPR assessed on day 1 and at day 2 24 h.
