# Supplementary material for: Acetylsalicylic acid in critically ill patients: a cross‐sectional and a randomized trial
Source: Eur J Clin Invest. 2017 Jun 20;47(7):504–12. doi: 10.1111/eci.12771 (PMC5519937; doi:10.1111/eci.12771)
Supplement: Supplementary file 1 — Appendix S1. Methods [file ECI-47-504-s001.docx]

**Supplement: Methods**

**Whole blood aggregometry**

Whole blood aggregation was determined using the Multiple Electrode Aggregometry (MEA) on the Multiplate Analyzer (Dynabyte Medical).The system detects the electrical impedance change due to the adhesion and aggregation of platelets on two independent electrode-set surfaces in the test cuvette A 1:2 dilution of whole blood anti-coagulated with heparin and 0.9% NaCl was stirred at 37°C for 3 min in the test cuvettes, ADP (adenosine diphosphate, 6.4μM), or arachidonic acid (AA =0.5 mM) were added and the increase in electrical impedance was recorded continuously for 6 min [1]. The mean values of the two independent determinations are expressed in units (U: tenth of area under the curve). The reference values for the test are as follows: ADP 29–118 U [2] and AA 75–130 U (according to the manufacturer's information). A good reproducibility of MEA has been reported (<6% variability) [3].

No generally accepted cut-off value for AA-induced platelet aggregation to identify patients with HTPR under ASA treatment has been defined yet. We chose a cut-off of >30 U (arbitrary units) for our study. This is based on other trials and supported by data from a previous trial in healthy volunteers [4-7]. In this trial AA induced platelet aggregation of 10±8 U was detected after ASA intake [7].

**Platelet Function analyzer 100 (PFA-100):**

The PFA-100 (Dade Behring, Marburg, Germany) was used for measuring platelet function under high shear rates (5000-6000s^-1^) as described previously [8] (see also supplement).

Blood samples collected in 3.8% sodium citrate were used. The PFA-100 measures the time required for occlusion of the aperture by platelet plugs, which is defined as closure time (CT). The instrument aspirates a blood sample under constant vacuum from the sample reservoir through a capillary and microscopic aperture (147µm) cut into the membrane, which leads to high shear induced platelet plug formation [8]. The membrane is coated with collagen/epinephrine (CEPI) or collagen/ADP (CADP) [9]. The reference values for the CEPI-CT is 75-193 seconds (s) and 65-120s for CADP-CT [10]. Individual day-to-day variability was reported for CEPI-CT (9%) Published data have shown a satisfactory reproducibility of the test. Less than 2% of samples have shown a variation of more than 20% between the repeated measurements.

As a cut-off value for HTPR a closure time <193 s was recommended [9, 10].

**Pharmacokinetics**

Analyses were performed using an AB Sciex QTRAP 5500 (AB Sciex, Framingham, US) system. 0.1 mL of an internal standard working solution with 500 ng/mL D4-ASA (Toronto Research Chemicals Inc., Toronto, Canada), and Phenyl-^13^C_6_-SA (Sigma-Aldrich, St. Louis, US) in acetonitrile was added to 0.1 mL plasma. The precipitated samples were vortexed and centrifuged at 2400 x g. After a 1:2 dilution of the supernatant with 0.1% formic acid, 5 μL were injected into the LC-MS/MS using the electrospray ionization in negative mode. Chromatographic separation was achieved on a Kinetex Reversed Phase C18 column (particle size 2.6 µm, 50 mm x 3 mm, Phenomenex, Torrance, US) using a mixture of 0.1% formic acid/acetonitrile, (60/40, v/v) at a flow rate of 0.4 mL/min. The transitions m/z 178.9 to m/z 137.0 for ASA, m/z 182.9 to m/z 141.0 for D4-ASA, m/z 136.9 to m/z 93.0 for the SA, and m/z 143.0 to m/z 99.1 for Phenyl-^13^C_6_-SA were monitored in multiple reaction-monitoring mode.

A calibration curve (25, 50, 100, 250, 500, 1000, 2500, 5000 ng/mL) with plasma samples was constructed for ASA and SA (both Sigma-Aldrich, St. Louis, US), respectively.

**Statistics**:

A repeated measures ANOVA was performed to compare repeatedly measured parameters. Since three groups were compared in this analysis we corrected for multiple testing by applying the Bonferroni procedure.

The assumption of sphericity was tested by Mauchly’s test for sphericity and tests were chosen accordingly. Multivariate tests included Pillai-Spur, Wilks-Lambda, Hotelling-Spur and Roy’s largest root, whereas univariate tests for within group differences over time, if sphericity was not assumed included Greenhouse Geisser test or Huynh-Feldt test, depending on the epsilon (>0.75 Greenhouse Geisser test, <0.75 Huynh-Feldt test)).

**References**

1. Sibbing D, Braun S, Jawansky S, Vogt W, Mehilli J, Schomig A, Kastrati A, von Beckerath N: **Assessment of ADP-induced platelet aggregation with light transmission aggregometry and multiple electrode platelet aggregometry before and after clopidogrel treatment.** *Thromb Haemost* 2008, **99:**121-126.

2. Mueller T, Dieplinger B, Poelz W, Calatzis A, Haltmayer M: **Utility of whole blood impedance aggregometry for the assessment of clopidogrel action using the novel Multiplate analyzer--comparison with two flow cytometric methods.** *Thromb Res* 2007, **121:**249-258.

3. Toth O, Calatzis A, Penz S, Losonczy H, Siess W: **Multiple electrode aggregometry: a new device to measure platelet aggregation in whole blood.** *Thromb Haemost* 2006, **96:**781-788.

4. Jambor C, Weber CF, Gerhardt K, Dietrich W, Spannagl M, Heindl B, Zwissler B: **Whole blood multiple electrode aggregometry is a reliable point-of-care test of aspirin-induced platelet dysfunction.** *Anesth Analg* 2009, **109:**25-31.

5. Jastrzebska M, Chelstowski K, Wodecka A, Siennicka A, Clark J, Nowacki P: **Factors influencing multiplate whole blood impedance platelet aggregometry measurements, during aspirin treatment in acute ischemic stroke: a pilot study.** *Blood Coagul Fibrinolysis* 2013, **24:**830-838.

6. Bolliger D, Filipovic M, Matt P, Tanaka KA, Gregor M, Zenklusen U, Seeberger MD, Lurati Buse G: **Reduced aspirin responsiveness as assessed by impedance aggregometry is not associated with adverse outcome after cardiac surgery in a small low-risk cohort.** *Platelets* 2016, **27:**254-261.

7. Hobl EL, Schmid RW, Stimpfl T, Ebner J, Jilma B: **Absorption kinetics of low-dose chewable aspirin--implications for acute coronary syndromes.** *Eur J Clin Invest* 2015, **45:**13-17.

8. Jilma B: **Platelet function analyzer (PFA-100): a tool to quantify congenital or acquired platelet dysfunction.** *J Lab Clin Med* 2001, **138:**152-163.

9. Fuchs I, Spiel AO, Frossard M, Derhaschnig U, Riedmuller E, Jilma B: **Platelet hyperfunction is decreased by additional aspirin loading in patients presenting with myocardial infarction on daily aspirin therapy.** *Crit Care Med* 2010, **38:**1423-1429.

10. Jilma-Stohlawetz P, Hergovich N, Homoncik M, Dzirlo L, Horvath M, Janisiw M, Panzer S, Jilma B: **Impaired platelet function among platelet donors.** *Thromb Haemost* 2001, **86:**880-886.
