# Supplementary figures and images for: Acetylsalicylic acid in critically ill patients: a cross‐sectional and a randomized trial
Source: Eur J Clin Invest. 2017 Jun 20;47(7):504–12. doi: 10.1111/eci.12771 (PMC5519937; doi:10.1111/eci.12771)

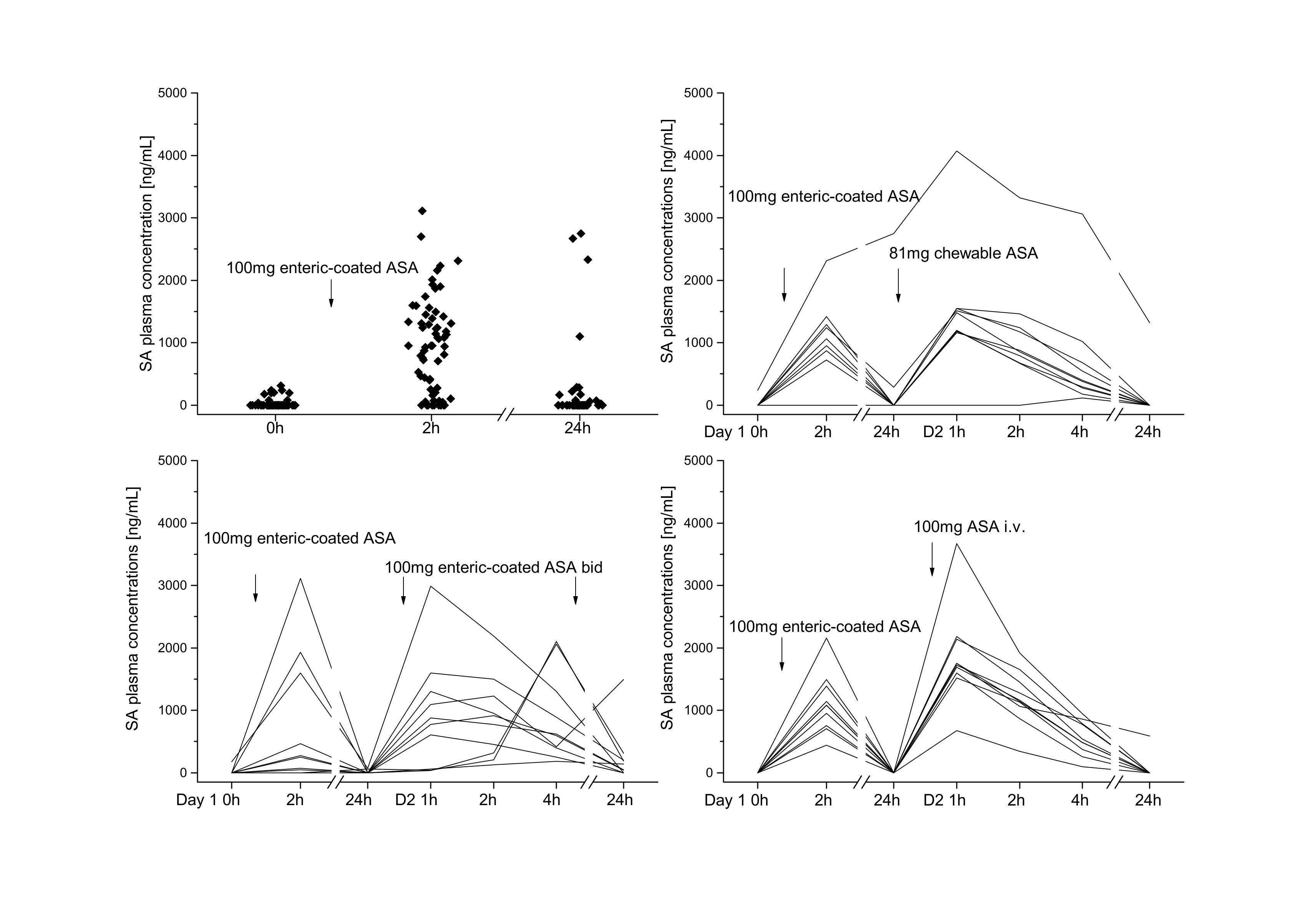

Supplement: Supplementary file 2 — Figure S1. Individual plasma concentrations of acetylsalicylic acid and salicylic acid in ng/mL after intake of 100 mg enteric‐coated acetylsalicylic acid (left upper panel), 81 mg chewable acetylsalicylic acid (right upper panel), 100 mg enteric‐coated acetylsalicylic acid (left lower panel) and infusion of 100 mg acetylsalicylic acid (right lower panel). [file ECI-47-504-s002.jpg]
