# Supplementary material for: Acetylsalicylic acid in critically ill patients: a cross‐sectional and a randomized trial
Source: Eur J Clin Invest. 2017 Jun 20;47(7):504–12. doi: 10.1111/eci.12771 (PMC5519937; doi:10.1111/eci.12771)
Supplement: Supplementary file 3 — Table S1. Arachidonic Acid‐induced platelet aggregation using whole blood aggregometry after alternative ASA treatments. [file ECI-47-504-s003.docx]

Table S1. Arachidonic Acid-induced platelet aggregation using whole blood aggregometry after alternative ASA treatments.

|  | Treatments | | |
| --- | --- | --- | --- |
| Timepoints | 100mg enteric-coated ASA | | |
| Day 1 0h | 64±30 | 68±35 | 72±37 |
| Day 1 2h | 53±25 | 51±28 * | 70±42 |
| Day 1 24h | 70±39 | 79±46 | 72±30 |
|  | 81mg chewable ASA | 100mg ASA i.v. | 100mg enteric- coated ASA bid |
| Day 2 1h | 59±37 | 38±21 ** | 72±39 |
| Day 2 2h | 53±32* | 49±31* | 74±57 |
| Day 2 4h | 53±36* | 47±21* | 62±27* |
| Day 2 24h | 79±41 | 57±41* | 52±27* |

Table S1. Arachidonic Acid-induced platelet aggregation using whole blood aggregometry after intake of 81mg chewable ASA, 100mg ASA i.v. or 100mg enteric-coated ASA bid Statistical testing was performed vs. baseline value. Presented are means ± standard deviations

*p<0.05 **p≤0.005
