# Supplementary material for: Acetylsalicylic acid in critically ill patients: a cross‐sectional and a randomized trial
Source: Eur J Clin Invest. 2017 Jun 20;47(7):504–12. doi: 10.1111/eci.12771 (PMC5519937; doi:10.1111/eci.12771)
Supplement: Supplementary file 4 — Table S2. Acetylsalicylic acid (ASA) and Salicylic acid (SA) plasma concentrations after different ASA treatments. [file ECI-47-504-s004.docx]

Table S2. Acetylsalicylic acid (ASA) and Salicylic acid (SA) plasma concentrations after different ASA treatments

|  | Treatments | | | | | | | |
| --- | --- | --- | --- | --- | --- | --- | --- | --- |
| Time-points | 100mg enteric-coated ASA (n=66) | | 81mg chewable (n=10) | | 100mg i.v. (n=10) | | 100mg enteric-coated ASA bid (n=10) | |
|  | *ASA* | *SA* | *ASA* | *SA* | *ASA* | *SA* | *ASA* | *SA* |
| 0h | 0 (0-64) | 0 (0-310) | 0 (0-247) | 0 (0-2750) | 0 (0-62) | 0 (0) | 0 (0) | 0 (0-72) |
| 1h | n.d. | n.d. | 202 (0-720) | 1340 (0-4070) | 220^§^ (110-607) | 1735* (673-3670) | 143 (0-255) | 876 (37-2990) |
| 2h | 63 (0-301) | 949 (0-3110) | 53 (0-162) | 864 (0-3320) | 67 (41-144) | 1150 (345-1920) | 87 (40-326) | 827 (127-2190) |
| 4h | n.d. | n.d. | 0 (0-75) | 388 (112-3060) | 0 (0-68) | 511 (96-944) | 41 (0-159) | 593 (185-2100) |
| 24h | 0 (0-1270) | 0 (0-2750) | 0 (0-133) | 0 (0-1370) | 0 (0-315) | 0 (0-738) | 0 (0-461) | 44 (0-1490) |

Table S2. Plasma Concentrations of acetylsalicylic acid and salicylic acid after intake of various ASA treatments are presented (Medians ± interquartile ranges).

n.d.=not done
