# Supplementary material for: Acetylsalicylic acid in critically ill patients: a cross‐sectional and a randomized trial
Source: Eur J Clin Invest. 2017 Jun 20;47(7):504–12. doi: 10.1111/eci.12771 (PMC5519937; doi:10.1111/eci.12771)
Supplement: Supplementary file 5 — Table S3. HTPR assessed on day 1 and at day 2 24 h. [file ECI-47-504-s005.docx]

**Table S3.** HTPR assessed on day 1 and at day 2 24h

| Treatment | 100mg ASA i.v. (n=10) | 100mg enteric-coated ASA bid (n=10) | 81mg chewable ASA (n=10) |
| --- | --- | --- | --- |
| Multiplate | | | |
| HTPR Day 1 | 10 | 10 | 10 |
| HTPR Day 2 24h | 6 | 8 | 9 |
| PFA-100 | | | |
| HTPR Day 1 | 4 | 6 | 3 |
| HTPR Day 2 24h | 1 | 2 | 5 |

Table S3. Frequency of HTPR on day 1 (standard treatment) and day2 24h (alternative treatments). Ten patients with HTPR classified by MEA results were randomized to receive 100mg ASA i.v. , 100mg enteric-coated ASA bid or 81mg chewable ASA. Results are absolute numbers of patients with HTPR on trial day 1 and on trial day 2 at 24h.
